# Characterization of an isolated lactase enzyme produced by *Bacillus licheniformis* ALSZ2 as a potential pharmaceutical supplement for lactose intolerance

**DOI:** 10.3389/fmicb.2023.1180463

**Published:** 2023-09-13

**Authors:** Alaa A. Amin, Zakia A. Olama, Safaa M. Ali

**Affiliations:** ^1^Botany and Microbiology Department, Faculty of Science, Alexandria University, Alexandria, Egypt; ^2^Nucleic Acid Research Department, Genetic Engineering and Biotechnology Research Institute, the City of Scientific Research and Technological Applications, Alexandria, Egypt

**Keywords:** lactase, Plackett-Burman, Box-Benken, characterization, purification

## Abstract

**Introduction:**

Lactose intolerance is a widespread problem that affects people of many different races all over the world. The following pharmacological supplements can improve the lives of those who suffer from this issue.

**Methods:**

This work focused on lactase producer isolation and statistical design (Plackett–Burman, and BOX–Behnken) to maximize the effectiveness of environmental factors. A lactase-producing bacterium was chosen from a discovery of 100 strains in soil that had previously been polluted with dairy products. Plackett-Burman investigated fifteen variables.

**Results:**

The most critical variables that lead to increased lactase synthesis are glucose, peptone, and magnesium sulfate (MgSO_4_). The ideal process conditions for the creation of lactase yield among the stated variables were then determined using a BOX-Benken design. To establish a polynomial quadratic relationship between the three variables and lactase activity, the Box–Behnken design level was used. The EXCEL-solver nonlinear optimization technique was used to predict the best form for lactase production. The ideal temperature and pH levels have been determined, both before and after the lactase purification process, to achieve the highest performance of isolated lactase.

**Conclusion:**

According to this study, *Bacillus licheniformis* is a perfect supply of the lactase enzyme (β -Galactosidase), It can be used as a product to assist people who have health issues due to lactose intolerance.

## 1. Introduction

Enzymes are active proteins that function as biochemical catalysts (Piumetti and Illanes, 2022). These are biomolecules that are required for both the synthesis and breakdown reactions of living organisms (Aggarwal et al., 2021). The enzymes known as β-galactosidases (EC 3.2.1.23), sometimes known as lactases, hydrolyze lactose (the most prevalent milk sugar) into glucose and galactose (Liburdi and Marco, 2022). Although these enzymes are accessible in a range of biological systems, such as plants or microorganisms, they are currently only available commercially from yeasts, molds, and bacteria (Saqib et al., 2017; Martarello et al., 2019). Although these enzymes from various bacteria have varied features, their specificity is fundamentally the same (Ramos and Malcata, 2011; Kocabaş et al., 2021). Each chain of the four polypeptides in the tetramer of β-galactosidase has 1,023 amino acids, and together they form five different structural domains (Sedzro et al., 2018; Swentowsky, 2021). One of these domains is the jellyroll barrel, while the others are fibronectin, b-sandwich, and a central domain with a TIM-type barrel that also serves as the active site. Tetramer subunits make up the core domain, which is catalytically active (Wang et al., 2021). The catalytic site is disabled when a tetramer is broken up into dimers. Those enzymes are active proteins and their function as biocatalysts were discovered by Cao et al. (2021). Similarly, the β-galactosidase amino acid sequence was done by Poch et al. (1992) and the structure was determined by Jacobson et al. (1994). This enzyme’s amino-terminal sequence contains a-peptide that engages in a-complementation and aids in the formation of the subunit interaction (Aydin and Coin, 2021; Xu et al., 2021). Numerous organisms, including fungi, plants, yeast, and bacteria, may produce lactase (Shaima'a and Rashid, 2017). In the industrial sector, bacterial strains offer a lot of promise for large-scale manufacturing. Lactase generated by bacteria was frequently used to hydrolyze lactose because of its ease of fermentation, high activity, and good stability (Movahedpour et al., 2022). β-Galactosidase is used in a variety of industries (Ureta et al., 2021). β-galactosidase is utilized to handle whey disposal concerns on a commercial scale in addition to manufacturing lactose-free goods for lactose-intolerant patients (Paulo, 2018). To avoid the problem of hygroscopic lactose crystallizing in food, β -galactosidase is employed to hydrolyze the lactose in frozen, concentrated sweets (Portnoy and Barbano, 2021). This treatment lowers the lactose level of milk so that lactose-intolerant people can drink it. Lack of lactase, a digestive enzyme that prevents the body from hydrolyzing lactose in meals, is the primary cause of lactose intolerance. About 75% of people worldwide have lactose intolerance, which significantly lowers their quality of life (Venkateswarulu et al., 2017; Peele et al., 2018). Probiotic supplements may be beneficial for those with lactase deficiency. The dairy industry used lactase-producing bacterial strains to cure milk-based products (Venkateswarulu et al., 2017). Stomach pain and abdominal distension, abdominal colic, diarrhea, and nausea are all signs of lactose intolerance (Shrestha et al., 2021). Lactase deficiency can be classified as primary, congenital, or secondary. Primary lactase insufficiency affects adults aged 2 to 20. A more prevalent variety is primary lactase deficiency, which is caused by a decrease in β-galactosidase production along the small intestine’s brush boundaries (lactase). The second kind of lactase deficit is a birth defect in lactase production, which is brought on by a genetic defect and is defined by patients having either little or nonexistent lactase enzyme at all. The third kind, sometimes referred to as secondary lactase deficit, is when there are inadequate levels of this enzyme as a result of a GI tract issue (Paulo, 2018; Szilagyi et al., 2019; Muzaffar et al., 2021).

The current study’s major purpose is to develop exploitation tactics for the eventual advantage of extracellular bacterial lactase production. The goal of this study was to uncover a novel approach to lactase manufacturing in a newly discovered lactase producer. Furthermore, the utilization of mathematical models was the main emphasis of the current investigation to optimize the growth conditions that result in the best lactase productivity from a newly discovered *Bacillus licheniformis ALSZ2*. To our knowledge, lag phase bacterial lactase production has not yet been documented in the literature; the current study’s findings are the first report of Egyptian lactase manufacturing that is significantly cost-effective.

## 2. Materials and method

### 2.1. Isolation and screening of lactase producers

Samples of diverse dairy products (12) (milk, Romy cheese, and karish cheese) were collected in a sterile dry container. For bacterial isolation and colony purification, an LB medium that was diluted to one-tenth of medium strength was used. For lactase production, 100 purified bacterial isolates were screened. Isolation of lactase-producing bacteria was achieved using an LB broth medium fortified with lactose substrate (5%) and 1% X-gal. Blue color after growth refers to lactase production.

#### 2.1.1. Rapid plate assay method

Qualitative identification of lactase producers was achieved using an LB agar medium containing 1 mM of IPTG (substrate of lactase) and X-gal (1%). The solidified agar plates were incubated in an inverted position at 37°C for 24 h. Isolated colonies that are blue (positive lactase producer) were subcultured in nutrient broth and incubated at 37°C and preserved for further studies (Petassi et al., 2020).

### 2.2. Molecular identification of the most potent producer

Fresh bacterial cells are used for DNA extraction using GeneJET Genomic DNA Purification Kit. Using standard primers (F: AGAGTTTGATCMTGGCTCAG and R: TACGGYACCTTGTTACGACTT) (Alkhafaje et al., 2019) intended to magnify a 1,500 base pair portion of the 16S rDNA region (Kim and Chun, 2014; Soliman et al., 2018), the 16S rDNA was amplified using PCR (polymerase chain reaction). The condition of the cyclical reaction was 4 min at 95°C and then 40 cycles of 40 s at 94°C, 50 s at 55°C, and 50 s at 72°C, monitored by an additional 10 min at 72°C. PCR reactions were run on an agarose gel, and the remaining mixture was purified for sequence (Alkhafaje et al., 2022).

#### 2.2.1. Phylogenetic analysis

To evaluate the DNA similarity of the obtained 16SrDNA sequence [by using a 3,130 X DNA Sequencer (Genetic Analyzer, Applied Biosystems, Hitachi, Japan)], phylogenetic analysis was conducted using the BLAST tool.^1^ Mega7 software was used to accomplish molecular phylogeny and multiple sequence alignment present in the database (NCBI). A neighbor-joining (NJ) tree and a maximum parsimony (MP) tree using bootstrapping were made using this alignment (Hassan et al., 2018; Shaaban et al., 2022).

### 2.3. Enzyme assay

The culture media were prepared, inoculated with lactase producers, and incubated for 24 h at 37°C. At the end of the incubation period, the fermented culture was centrifuged at 5000 rpm for 10 min at room temperature (at 25°C). 0.8 mL of the cell-free extract (crude enzyme) was incubated with 1.2 mL of 5% lactose at 37°C for 30 min. Enzyme lactase hydrolyses lactose into glucose and galactose, and this reaction was terminated by boiling the crude enzyme extract for 10 min. Glucose was then measured using a Glucose kit (Biosystem) at 540 nm (Amamcharla and Metzger, 2011). The quantity of enzyme necessary to yield 1 μg of glucose every minute under typical test circumstances was considered to be a single lactase unit.

### 2.4. Optimization of lactase producer using experimental design

#### 2.4.1. Plackett-Burman screening design

Using the Plackett-Burman experimental design, the effects of various medium compositions on the production of the lactase enzyme were investigated. Constructed using a Plackett-Burman matrix (Supplementary Table S1) (Ali et al., 2013; Sorour et al., 2020), a two-level factorial design was used to examine fifteen variables at two levels, −1 for the low value and + 1 for the high value. This permits the analysis of n − 1 variables with the fewest number of tests. To determine the relative significance of 15 elements or variables (MgSO_4_, Glucose, NaNO_3_, CaCl_2_, CuSO_4_, MnSO_4_, ZnSO_4_, FeSO_4_, KCL, NaHPO_4._12H_2_O, KH_2_PO_4_, K_2_HPO_4_, yeast extract (YE), Beef extract, and Peptone), an experimental design which includes a set of 20 experiments (trials) was used. The first-order model served as the basis for the Plackett-Burman design: *Y* = o + iXi, where *Y* represents the response (enzyme activity), o represents the model intercept, I represents the linear coefficient, and Xi represents the degree of an independent variable. Developing the free Essential Experimental Design program, data analysis, coefficient determination, and polynomial model reduction was performed on the lactase enzyme statistics.

#### 2.4.2. Box-Benken design

To discover the kind of response surface in the experiment and to decide the greatest circumstances for enzyme synthesis, a Box-Benken design (Ali et al., 2013; Sorour et al., 2020; Tamer et al., 2021) was adopted. The factorial design, which includes thirteen trials, was constructed to look into the most critical parameters that influence enzyme synthesis. Each variable was examined at three different levels, with low, moderate, and high values, respectively, denoted by −1, 0, and + 1 (Supplementary Table S2). A second-order polynomial equation was developed to determine the appropriate location and to connect the relationship between the independent components and the response for three parameters. The equation was:


$$\begin{array}{l} Y=\beta 0+\beta 1\mathrm{X1}+\beta 2\mathrm{X2}+\beta 3\mathrm{X3}+\beta 1\mathrm{2X}12+\beta 1\mathrm{3X}13\\ \;\;\;\;\;+\beta 2\mathrm{3X}23+\beta 1\mathrm{1X}12+\beta 2\mathrm{2X}22+\beta 3\mathrm{3X}32 \end{array}$$

Where Y is the expected response, 0 is the design constant, X1, X2, and X3 are the independent factors, 12–13 and 23 are cross-product constants, 11–22 and 33 are quadratic constants, and 12–13 and 23 are coefficients of the cross product, respectively, and 11–22 and 33 are quadratic coefficients, and 12–13 and 23 are cross product coefficients, respectively. The experimental data were subjected to a regression analysis using Microsoft Excel 2010. The constant of determination, R2, was employed to explain the polynomial model equation’s grade of fit (Ali et al., 2013; Sorour et al., 2020). The tests were repeated three times, with the average results presented.

### 2.5. Purification of lactase enzyme

The crude enzyme was purified using the column chromatography (gel filtration) technique. Multiple columns with different pour sizes were applied to detect the molecular weight and extract the target enzyme (Amicon system). The system combines downstream sample concentration and buffer exchange with affinity-based spin column purification. The tool eliminates the requirement for numerous centrifugation processes and has a large reservoir that can hold a variety of sample quantities. For simultaneous elution, concentration, and extremely effective diafiltration (>99%) in a single spin, the included Amicon® Ultra filter (Philippines - Merck Millipore Division, an affiliate of Merck KGaA, Darmstadt, Germany) was attached. According to the protein cut-off different filters were used (UFC8010, UFC8020, UFC8030, UFC8050, UFC8070, and UFC8100 for 10, 20, 30, 50, 70, and 100 KD respectively). The concentrated protein fraction was very carefully layered on top of the pre-equilibrated and stabilized falcon tubes. The tubes were then centrifuged under cooling (4°C) for 20 min at 8000 rpm, and the upper layer was transferred to the tube with the cut-off (10, 20, 30, 50, 70, and 100 KD) as presented in the supplementary file. Protein content was estimated in each of the filter cut-offs, using the bovine serum albumin (BSA) standard curve The enzyme activity of each of the resulting various molecular weight proteins was then determined (Patel et al., 2017).

#### 2.5.1. Characterization of purified and crude lactase

##### 2.5.1.1. Determination of optimum temperature

At pH 7, temperatures between 30–60°C were found to be the ideal temperature for the crude and purified enzyme activity.

##### 2.5.1.2. Obtaining the ideal pH for enzyme activity

In a trial to test the effect of pH level on crude and purified enzyme activities one at a time, different buffers, namely sodium phosphate buffer, Tris–HCl buffer, and potassium phosphate buffer with varied pH ranges (5.8–8), (6–8.5), and (5.8–8) respectively, were used.

### 2.6. The antibiotics sensitivity of ALSZ2

Seventeen antibiotic disks were tested, namely amikacin (AMK, 30 μg), amoxicillin-clavulanic acid (AMC, 20 μg/10 μg), ampicillin (AMP, 10 μg), aztreonam (ATM, 30 μg), cefepime (FEP, 30 μg), ceftazidime (CAZ, 30 μg), chloramphenicol (CHL, 30 μg), ciprofloxacin (CIP, 5 μg), clindamycin (CLI, 2 μg), clotrimazole (CC, 10 μg), erythromycin (ERY, 15 μg), gentamicin (GEN, 10 μg), imipenem (IPM, 10 μg), kanamycin (KAN, 30 μg), ticarcillin-clavulanic acid (TIM, 75 μg/10 μg), trimethoprim (TMP, 5 μg) and vancomycin (VAN, 30 μg). The prepared plates were incubated for 24 h at 37°C. The inhibition zone for each antibiotic was measured with a ruler. The indicator of antibiotic sensitivity has traditionally been the existence of a clear zone surrounding the disk. The diameter of the inhibitory zone was used to calculate the results of antibiotic sensitivity tests.

## 3. Results

In the beginning, many bacteria were isolated from different sources of dairy products, and then the isolates were compared to produce the required enzyme and determine the best one in terms of productivity.

### 3.1. Isolation and screening of lactase (β-galactosidase) producers

**Figure 1:**
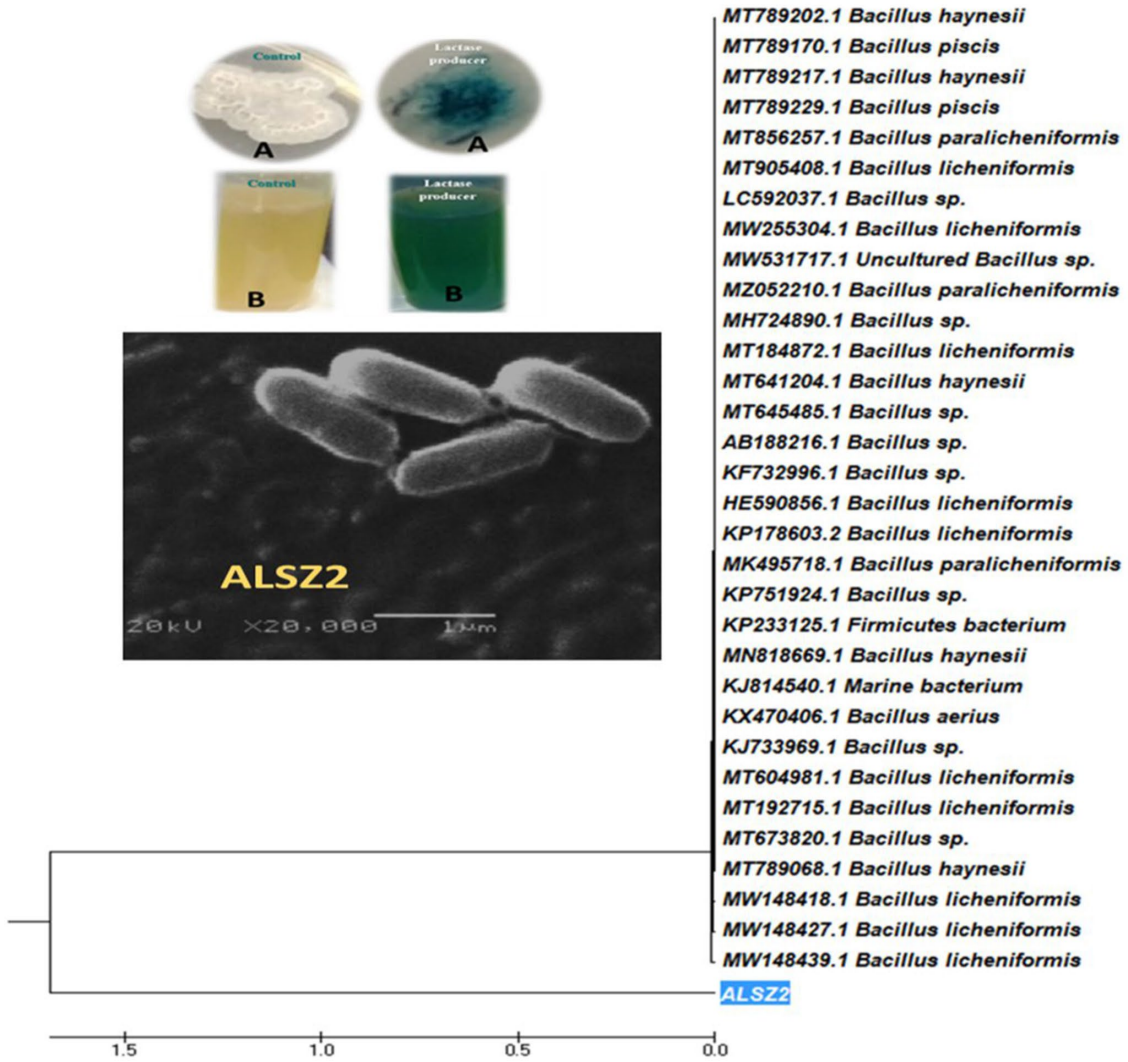
Phylogenetic tree of *Bacillus licheniformis* ALSZ2 based on 16S RNA sequence assessments. Detection of lactase production using colorimetric methods: **(A)** shows the negative control and positive lactase producer using solid media and **(B)** shows the negative control and positive lactase producer using liquid media.

In a screening process for lactase enzyme-producing bacteria, 100 isolates were collected from dairy product samples and examined for enzyme production. Lactase-producing bacteria were screened qualitatively on X-Gal agar plates (Figure 1A) and nutrient broth media containing X-Gal (Figure 1B) at 37°C. Thirteen out of 100 bacterial isolates showed a variance in enzyme activity. Isolate ALSZ2 was the most promising lactase producer and was related for further work.

### 3.2. Identification of the selected bacterial isolates

The most promising isolate with the code ALSZ2 was identified using PCR-amplified 16S rDNA genes. Molecular identification and gene bank sequence isolate (ALSZ2) was *Bacillus* genus, with a 96.66 present similarity to *B. licheniformis*, according to the findings. The Mega 7 program was used to generate a phylogenetic tree, showing that isolate (ALSZ2) is more strongly related to *B. licheniformis* bacterium (acc.: MW148439.1), shown in Figure 1.

### 3.3. The antibiotic sensitivity of the selected bacterial isolates

The antibiotics amikacin, amoxicillin-clavulanic acid, ampicillin, aztreonam, cefepime, ceftazidime, chloramphenicol, ciprofloxacin, clindamycin, clotrimazole, erythromycin, gentamicin, imipenem, kanamycin, ticarcillin-clavulanic acid, trimethoprim, and vancomycin were all very sensitive to *B. licheniformis* ALSZ2. The strain was resistant to ampicillin and cefepime.

### 3.4. Improving the nutritive necessities upsetting *Bacillus licheniformis* ALSZ2 lactase production using multifactorial statistical design: Plackett-Burman and Box–Behnken designs

#### 3.4.1. Selection of important variables upsetting lactase production using a Plackett-Burman design

The “two-phase” optimization strategy was used to apply the statistical design. The initial stage was to determine the relative relevance of the various components in the culture media, as well as the levels of variables that have a major impact on lactase synthesis. The trials were then verified to validate the results under precise, optimal experimental settings.

**Figure 2:**
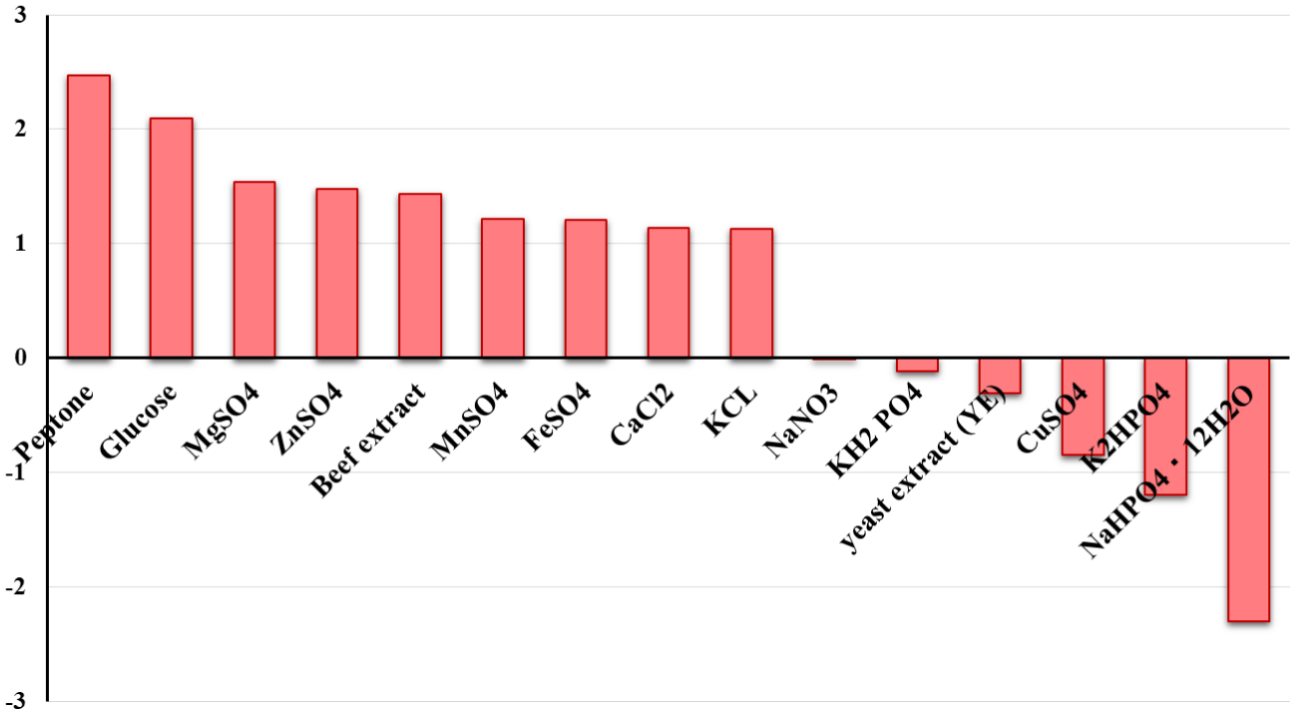
The main effect of different variables on *B. licheniformis* ALSZ2 lactase production based on Plackett-Burman design results.

Plackett-Burman design for twenty trials with two concentration levels for fifteen different variables (Supplementary Table S1) was conducted based on the experimental matrix shown in Table 1. The corresponding results were summarized in Table 2. The Plackett-Burman design studies’ results revealed a wide range of variations. To assess variables that affect lactase production, the Plackett-Burman statistical design was employed (*B. licheniformis* ALSZ2). Calculations and graphical representations of the primary impacts of the investigated factors on lactase activity are shown in Figure 2. A major effect value with a positive sign suggests that a variable’s high concentration is close to its optimal level, whereas the main effect value with a negative sign indicates that a variable’s low concentration is close to its optimal level (Figure 2 and Table 3). It was found that Peptone, MgSO_4_, and Glucose have the greatest positive effect on the production, followed by ZnSO_4_, Beef extract, KCl, FeSO_4_, MnSO_4_, and CaCl_2_. Whereas, NaHPO_4_ has the most negative effect, followed by K_2_HPO_4_, CuSO_4_, yeast extract, KH_2_PO_4_, and NaNO_3_ in order.

**Table 1:** Plackett-Burman design matrix for fifteen variables with coded levels for *Bacillus licheniformis* ALSZ2 lactase optimization.

Trail no.	Variables															Lactase activity (U/mL)
	x1	x2	x3	x4	x5	x6	x7	x8	x9	x10	x11	x12	x13	x14	x15	
1	1	−1	−1	1	1	1	1	−1	1	−1	1	−1	−1	−1	−1	1.75
2	−1	−1	1	1	1	1	−1	1	−1	1	−1	−1	−1	−1	1	0
3	−1	1	1	1	1	−1	1	−1	1	−1	−1	−1	−1	1	1	6.125
4	1	1	1	1	−1	1	−1	1	−1	−1	−1	−1	1	1	−1	4
5	1	1	1	−1	1	−1	1	−1	−1	−1	−1	1	1	−1	−1	0
6	1	1	−1	1	−1	1	−1	−1	−1	−1	1	1	−1	−1	1	5.25
7	1	−1	1	−1	1	−1	−1	−1	−1	1	1	−1	−1	1	1	0
8	−1	1	−1	1	−1	−1	−1	−1	1	1	−1	−1	1	1	−1	0
9	1	−1	1	−1	−1	−1	−1	1	1	−1	−1	1	1	−1	−1	0
10	−1	1	−1	−1	−1	−1	1	1	−1	−1	1	1	−1	−1	1	2.5
11	1	−1	−1	−1	−1	1	1	−1	−1	1	1	−1	−1	1	1	2.75
12	−1	−1	−1	−1	1	1	−1	−1	1	1	−1	−1	1	1	1	1.5
13	−1	−1	−1	1	1	−1	−1	1	1	−1	−1	1	1	1	1	1.125
14	−1	−1	1	1	−1	−1	1	1	−1	−1	1	1	1	1	−1	1.75
15	−1	1	1	−1	−1	1	1	−1	−1	1	1	1	1	−1	1	0
16	1	1	−1	−1	1	1	−1	−1	1	1	1	1	−1	1	−1	0
17	1	−1	−1	1	1	−1	−1	1	1	1	1	−1	1	−1	1	2
18	−1	−1	1	1	−1	−1	1	1	1	1	−1	1	−1	1	−1	0.125
19	−1	1	1	−1	−1	1	1	1	1	−1	1	−11	1	−1	−1	3.625
20	1	1	−1	−1	1	1	1	1	−1	1	−1	1	−1	−1	−1	1.375

**Table 2:** Statistical analysis of Plackett–Burman design showing *p*-values, *t* stat, standard error, and coefficient values for each variable on *B. licheniformis* ALSZ2.

	Intercept	Coefficients	Standard error	*t* Stat	*p*-value
		1.69375	0.331699	5.106285	0.006952
MgSO_4_	x1	0.775413	0.504984	1.535519	0.199455
Glucose	x2	0.86785	0.414914	2.091639	0.104637
NaNO_3_	x3	−0.00385	0.410797	−0.00938	0.992968
CaCl_2_	x4	0.461644	0.407376	1.133214	0.320442
CuSO_4_	x5	−0.34818	0.412497	−0.84408	0.446148
MnSO_4_	x6	0.493331	0.406293	1.214225	0.291444
ZnSO_4_	x7	0.67716	0.459307	1.474306	0.214413
FeSO_4_	x8	0.516835	0.428567	1.20596	0.294286
KCL	x9	0.519413	0.459307	1.130862	0.321323
NaHPO_4_・12H_2_O	x10	−0.93466	0.406293	−2.30046	0.082897
KH_2_ PO_4_	x11	−0.04708	0.412497	−0.11414	0.914629
K_2_HPO_4_	x12	−0.48765	0.407376	−1.19706	0.297376
Yeast extract (YE)	x13	−0.1281	0.410797	−0.31184	0.770739
Beef extract	x14	0.593642	0.414914	1.43076	0.22574

**Table 3:** Box–Behnken design matrix and results for the three most significant variables that affected *B. licheniformis* ALSZ2 lactase production.

Trail	MgSO_4_ (x1)	Glucose (x2)	Peptone (x3)	Lactase activity (IU)	Predicted IU lactase
1	0	−1	−1	7	6
2	0	+1	−1	6	7
3	0	−1	+1	7.5	7.25
4	0	+1	+1	7.25	7.5
5	−1	−1	0	8	7.75
6	−1	+1	0	8.75	8
7	+1	−1	0	8	8
8	+1	+1	0	9	8
9	−1	0	−1	8	8.75
10	−1	0	+1	7.75	8.75
11	+1	0	−1	9.25	9
12	+1	0	+1	8.75	9.25
13	0	0	0	13	13

##### 3.4.1.1. Verification of the model

Variables with negative main effect values were used as (−1) coded values, while variables with positive main effect values were used as (+1) coded values. Supplementary Table S3 shows the lactase activity variations X1, X2, X3, X4, X5, X6, X10, and X19. Considering the data gathered from the outcomes of the Plackett-Burman experiment, the following composition (g/L) is expected to be near to the optimal: Beef extract, 1; MgSO_4_, 10; Glucose, 75; NaNO_3_, 30; Yeast extract, 100; CaCl_2_, 1; CuSO_4_, 0; MnSO_4_, 10; ZnSO_4_, 15; FeSO_4_, 20; KCl, 1; NaHPO_4_.12H_2_O, 10; KH_2_PO_4_,10; K_2_HPO_4_, 0; Peptone, 25. The medium was reformed to a pH of 7 and the flasks were incubated at 37°C for 24 and 48 h, respectively. A verification experiment was done to estimate the reliability of the Plackett-Burman screening test.

#### 3.4.2. Optimization of medium composition by Box–Behnken design

To validate the precision of the variables identified by the Plackett-Burman design using the Box–Behnken design experimental strategy, Response Surface Methodology was used. The following three tiers of the three crucial factors were looked into: −1, 0, and + 1.

##### 3.4.2.1. Box–Behnken design for *Bacillus licheniformis* ALSZ2

**Figure 3:**
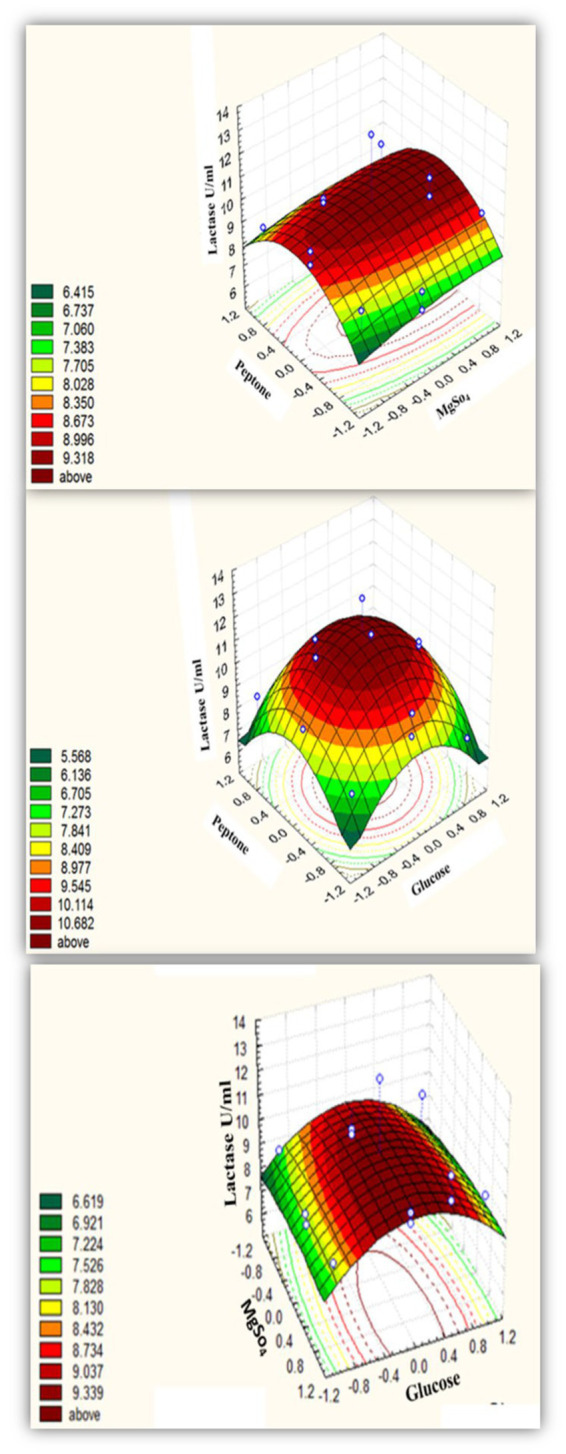
Three-dimensional response surface representing *B. licheniformis* ALSZ2 lactase enzyme as affected by culture conditions.

According to the Box–Behnken design, Supplementary Table S2 shows alternative combinations of the three essential variables. All assemblies had the same concentration of the remaining components as the Plackett-Burman pre-optimized medium. The Box–Behnken experimental design’s results are summarized in Table 3 and the output of the surface plots formula is in (Figure 3). The optimal condition was determined by examining the connection between the individual variables and the lactase activity response. Table 3 shows the design matrix. The design indicated the optimum environmental factors as predictable lactase activity of 13.01754 U/mL, even though 13 U/mL of activity existed under ideal circumstances. As a consequence, the accuracy grade of 99.8% was used to evaluate the power of the perfect matrix under the following ideal circumstances. The medium composition contained g/L: MgSO_4_, 10.5; Glucose, 75.4491; NaNO_3_, 30; CaCl_2_, 1; CuSO_4_, 0; MnSO_4_, 10; ZnSO_4_, 15; FeSO_4_, 20; KCl, 1; NaHPO_4_.12H_2_O, 10; KH_2_PO_4_,10; K_2_HPO_4_, 0; Yeast extract, 100; Beef extract, 1; Peptone, 25.249 at pH 7, and the bacteria were grown in a rotatory shaker set to 37°C and 200 rpm, for 24 h.

### 3.5. Purification of lactase enzyme

The cell-free extract was subjected to purification using an Amicon system to achieve the best possible result of enzyme activity with different protein molecular weight (MW) cut-offs (100, 50, 30, and 10 kDa). *Bacillus licheniformis* ALSZ2 lactase was detected in a 30–50 kDa cut-off filter.

### 3.6. Effect of temperature on lactase activity (crude and purified)

**Figure 4:**
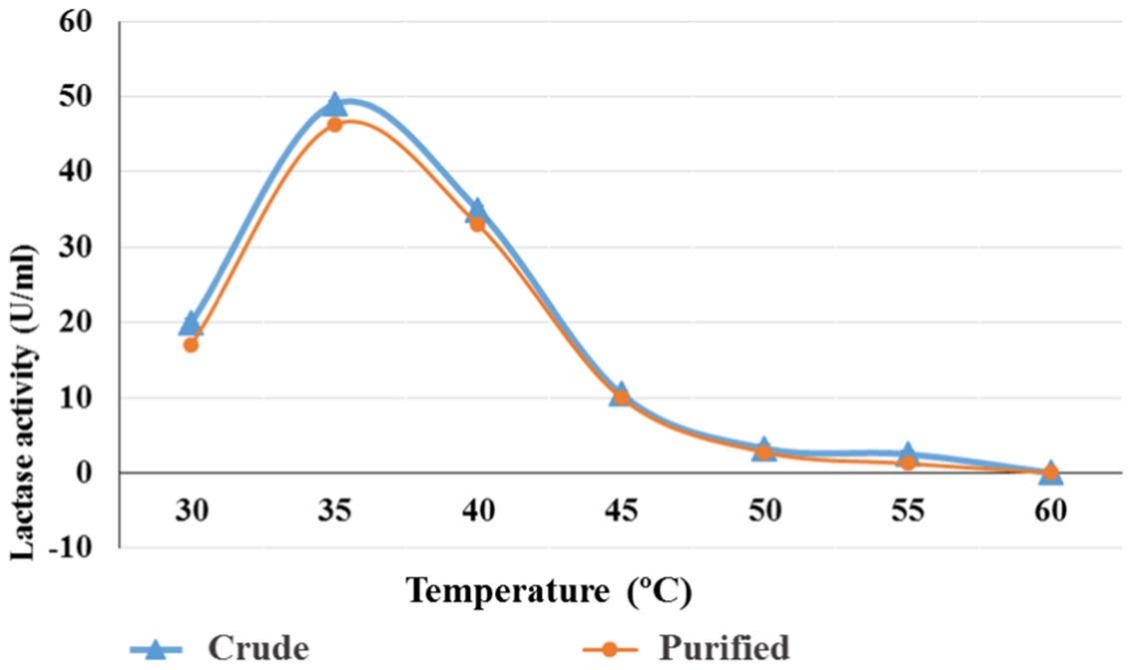
Optimum temperature for crude and purified lactase (30–50 kDa) from *B. licheniformis* AlSZ2.

*Bacillus licheniformis* ALSZ2 lactase (crude & purified) was exposed to seven different temperatures to ascertain the enzyme’s optimum temperature (30, 35, 40, 45, 50, 55, and 60°C Figure 4). Results in Figure 4 revealed that the perfect temperature for maximum enzyme activities (crude & purified) was found to be 35°C with 49 and 46.25 U/mL (30–50 kDa) respectively. Lactase synthesis reached a maximum of 90.05 IU at a temperature of 35°C.

### 3.7. Effect of hydrogen ion concentration on the enzyme activity (crude and purified)

**Figure 5:**
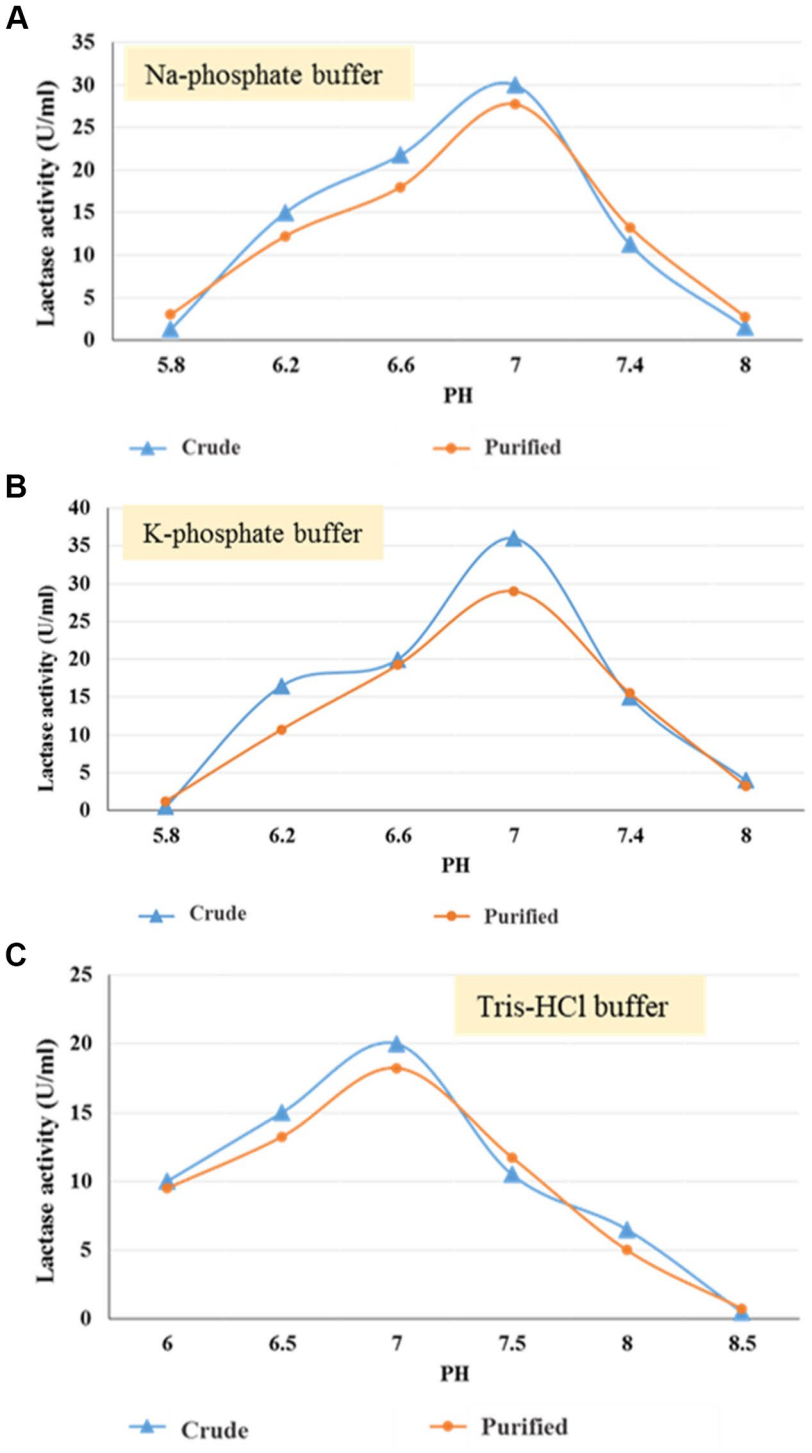
Optimum pH using Na-phosphate **(A)**, K-phosphate **(B)**, and Tris–HCl **(C)** buffer for crude and purified (30–50 kDa) lactase from *B. licheniformis* ALSZ2.

To determine the ideal pH for enzyme activity, three different buffers have been used, namely sodium phosphate, potassium phosphate, and Tris–HCl, each with a distinct pH concentration. Sodium phosphate and potassium phosphate have a pH range of 5.8–8, while Tris–HCl buffer has a pH range of 6–8.5. Results in Figures 5A–C indicated that the optimum enzyme (crude & purified) activities were 7 with all the detected buffers with enzyme activity ranging from 30 & 27.75, 36 & 29, and 20 & 18.25 U/mL, respectively.

### 3.8. Economic comparison between produced lactase and international products

The need for lactase medical supplements is increasing every day worldwide. The frequency of usage differed significantly between countries. This study provides a promising lactase enzyme product as the initial stage for lactase medical supplements. Comparing AlSZ2 lactase with other products available in the international market, it was found that AlSZ2 lactase is considerably more cost-effective. Many other products are expensive and often unavailable in the Egyptian market. The lactase products’ cost (1,000 U) range from 5.5–8 $, due to the high cost of shipping rather than the real price of the lactase product, whereas 1,000 U of AlSZ2 lactase costs 1.2 $. Therefore, this study is of great importance to cheaply provide lactase, which leads to raising the economic value and aiding in the treatment of a prevalent digestive problem.

## 4. Discussion

The frequency of Middle Easterners who are lactose intolerant was 70%, with 68 percent of Egyptians affected (Silanikove et al., 2015). Therefore, this study seeks to isolate bacteria capable of producing lactase that can be accessed cheaply.

The amount of carbon in the growth media is critical for bacteria to produce extracellular lactase. Lactase biosynthesis is regulated by carbon availability in various bacteria (Konsoula and Liakopoulou-Kyriakides, 2007; Alazzeh et al., 2009; Maischberger et al., 2010; Akcan, 2018). Based on the experimental matrix, a Plackett-Burman design (Karlapudi et al., 2018; Sorour et al., 2020) was carried out for twenty trials with two concentration levels for fifteen distinct variables (Supplementary Table S1). When using response surface approaches, Plackett and Burman Design is a useful tool for assessing the effects of process factors on yield. It can significantly lower the number of trials that must be repeated in later optimization investigations (Ekpenyong et al., 2017).

In previous research (Manera et al., 2008), lactose was found to be an excellent substrate when used in quantities more than 28.2 g per liter, while Anumukonda and Prabhakar (2010) discovered a 1.5 percent improvement in lactase synthesis in the optimized medium. Another study using solid-state fermentation with *Aspergillus terreus* NFCCI 1840 found that supplementing the cultured medium with 2.97, 2.88, and 2.67 g per liter of ammonium sulfate, lactose, and magnesium sulfate, respectively, increased lactase production by 2.8 times when compared to the standard basal medium (Al-Jazairi et al., 2015).

Ca^2+^ and Mg^2+^ increased enzyme activity in *Pediococcus acidilacti*, *Lactobacillus acidophilus*, and *Bacillus* sp. (Ustok et al., 2010; Carevic et al., 2015; Chanalia et al., 2018). Magnesium is required for the catalytic activity and stability of β -galactosidase. MgSO_4_ at a concentration of 0.1 percent, Ahmed, et al. (Ahmed et al., 2016) found that *Lactobacillus* sp. KLSA 22 produced the highest amount of β -galactosidase. Nitrogen sources are the most essential secondary energy molecules for bacterial growth and metabolism. The type of these compounds and the amounts used can either promote or prevent the growth of enzymes (Sharma and Singh, 2014). Sources of nitrogen’s effect on *L. casei*MB2’s ability to synthesize galactosidase is important. Peptone supplementation raised the enzyme activity of *L. casei*MB2 enzyme production medium to 126.24 IU/mL (specific activity of 315.60 IU/mg). Conversely, urea, sodium chloride, sodium nitrate, ammonium nitrate, and beef extract all showed a reduction in enzyme activity (Heena and Nivedita, 2020).

Numerous separation methods, such as membrane-based separation, ion exchange membrane chromatography, gel permeation chromatography, zinc chloride, protamine sulfate, and ammonium sulfate precipitation, have been investigated for the purification of beta-galactosidase from crude extract (Najer et al., 2022). β - Galactosidases with a range of molecular weights have been discovered in plant sources using the Amicon membrane system with a 100 to 30 kDa cut-off (Aulitto et al., 2021). Five enzymes with molecular weights of 87, 87, 87, 73, and 45 kDa have been recognized (Ajay et al., 2013).

The synthesis of enzymes grew continuously with increasing temperatures up to 35°C and then dropped (Ahmed et al., 2016). Natarajan et al. (2012) found that *Bacillus thuringiensis* produced the most enzymes at a temperature of 35°C. 37°C was the optimal degree of heating during fermentation, based on how temperature affects the activity of enzymes for the highest β -galactosidase yield (Husain, 2010; Huang et al., 2021).

At pH 7, the greatest enzyme activity was observed (89.94 IU), which was also a good pH for *Lactobacillus* sp. KLSA 228 growth. However, *L. amylophilus* GV6 was shown to produce the most lactase when grown at pH 6.513. Lactase from *L. delbrueckii* spp. ATCC 11842 was found to have the highest activity when pH was 6.8 (Ahmed et al., 2016; Venkateswarulu et al., 2020). Natarajan et al. (2012), on the other hand, found that lactase synthesis was best at a pH of 7.

## 5. Conclusion

Exploration of bacterial species with unique lactase capacities is critical. As a result, the current study aimed to isolate the most potent extracellular lactase-producing bacteria from various dairy products in various Alexandria locations, as well as increase lactase productivity by a local isolate *Bacillus* strain ASZ using low-cost materials via a sequential optimization strategy. The findings indicated that the chosen isolate is *B. licheniformis* ALSZ2, which is employed as a lactase-producing model. Through a two-level Plackett–Burman design, peptone, lactose, and MgSO_4_ were chosen as investigated variables because of their strong positive influence on lactase efficiency. The lactase yield increased with the initial basal medium, allowing a quadratic polynomial model to be developed that links the relationship between all three factors and lactase production. In comparison to the un-optimized medium, the estimated ideal lactase activity was 13 U/mL, which was four times higher. Following that, the purification of experimental bacterial lactase productivity was done. When compared to comparable lactase products on the worldwide market, AlSZ2 lactase is significantly less expensive.

## Data availability statement

The data presented in this study are deposited in the NCBI database under accession number OR535141.

## Author contributions

SA conceived the study and funded the project. Experimental work, data analysis, and primary publication writing were all done in collaboration with AA, ZO, and SA. All authors contributed to the article and approved the submitted version.

## Conflict of interest

The authors declare that the research was conducted in the absence of any commercial or financial relationships that could be construed as a potential conflict of interest.

## Publisher’s note

All claims expressed in this article are solely those of the authors and do not necessarily represent those of their affiliated organizations, or those of the publisher, the editors and the reviewers. Any product that may be evaluated in this article, or claim that may be made by its manufacturer, is not guaranteed or endorsed by the publisher.

## Footnotes

^1^www.ncbi.nlm.gov/blast
