# Supplementary material for: Characterization of an isolated lactase enzyme produced by Bacillus licheniformis ALSZ2 as a potential pharmaceutical supplement for lactose intolerance
Source: Front Microbiol. 2023 Sep 13;14:1180463. doi: 10.3389/fmicb.2023.1180463 (PMC10535568; doi:10.3389/fmicb.2023.1180463)
Supplement: Supplementary file 1 [file Data_Sheet_1.pdf]

Supplementary file

Title: **Isolation, optimization, purification, and characterization of lactase enzyme produced by *Bacillus licheniformis* ALSZ2 as an initial stage of the manufacturing process of pharmaceutical supplements for lactose intolerance patients**

Table 1S

Placket-Burman design variables for screening of lactase production by *Bacillus licheniformis* ALSZ2

Variable code.	Variable	Variable level (g/L)	
		-1	+1
X1	$MgSO_4$	10	40
X2	Glucose	50	75
X3	$NaNO_3$	10	30
X4	$CaCl_2$	1	2
X5	$CuSO_4$	0	2
X6	$MnSO_4$	0	10
X7	$ZnSO_4$	0	15
X8	$FeSO_4$	0	20
X9	KCL	1	2
X10	$NaHPO_4 \cdot 12H_2O$	0	7
X11	$KH_2 PO_4$	0	10
X12	K_2HPO_4	0	20
X13	yeast extract (YE)	50	400
X14	Beef extract	1	100
X15	Peptone	25	50

Table 2S

The levels of variables in Box-Benken for optimization of *Bacillus licheniformis* ALSZ2 lactase production

**Variable code.**	**Variable**	**Variables level (g/L)**		
		**-1**	**0**	**+1**
X1	MgSO4	5	10	15
X2	Glucose	37.5	75	112.5
X15	Peptone	12.5	25	37.5

Table 3S

Verification experiment for *B. licheniformis* ALSZ2 lactase production on basal versus pre-optimization medium at 24 &48 hours

Trail no.	x1	x2	x3	x4	x5	x6	x7	x8	x9	x10	x11	x12	x13	x14	x15	IU 24hr.	IU 48hr.
1	-1	1	1	-1	-1	1	1	1	1	-1	1	-1	1	-1	-1	2	2.5
2	-1	1	1	-1	-1	1	1	1	1	-1	1	-1	1	-1	-1	1.1125	1.1625
3	-1	1	1	-1	-1	1	1	1	1	-1	1	-1	1	-1	-1	5	5.25
4	-1	1	1	-1	-1	1	1	1	1	-1	1	-1	1	-1	-1	3.125	3
5	-1	1	1	-1	-1	1	1	1	1	-1	1	-1	1	-1	-1	0.625	0.75
6	-1	1	1	-1	-1	1	1	1	1	-1	1	-1	1	-1	-1	4.5	4.875
10	-1	1	1	-1	-1	1	1	1	1	-1	1	-1	1	-1	-1	2.5	2.75

Amicon system used in the study

According to using different protein cut off

- For 100 KD:

UFC8100: Amicon® Ultra-4 Centrifugal Filter Unit, **Ultracel-100 regenerated cellulose membrane, 4 mL sample volume.**

Synonym(s):

- For 10 Kd:

UFC8010: Amicon® Ultra-4 Centrifugal Filter Unit

- For 20 Kd:

UFC8020: Amicon® Ultra-4 Centrifugal Filter Unit

- For 30 kd:

UFC8030: Amicon® Ultra-4 Centrifugal Filter Unit

- For 50 kd:

UFC8050: Amicon® Ultra-4 Centrifugal Filter Unit

- For 70 kd:

UFC8070: Amicon® Ultra-4 Centrifugal Filter Unit
